# The Orbitofrontal–Anterior Temporal Network Complements the Attention Network for Where to Look in Social Situations

**DOI:** 10.1523/ENEURO.0203-26.2026

**Published:** 2026-10-06

**Authors:** Nico Marek, Radha Nila Meghanathan, Stefan Pollmann

**Affiliations:** ^1^Department of Psychology, Otto-von-Guericke Universität Magdeburg, Magdeburg 39106, Saxony-Anhalt, Germany; ^2^Center for Brain and Behavioral Sciences, Otto-von-Guericke Universität Magdeburg, Magdeburg 39106, Saxony-Anhalt, Germany

**Keywords:** attention, eye movement, fMRI, frontal, social neuroscience, temporal

## Abstract

Human neuroimaging experiments have consistently found dorsal and ventral attention networks subserving spatial shifts of covert attention or eye movements. However, these experiments used abstract, artificial stimuli and clearly defined instructions about where to attend. This clearly is not an adequate model of eye movement control in natural environments full of semantic and social information. Here, we show that during movie viewing, activation of the dorsal and ventral attention networks was complemented by activation patterns in the orbitofrontal and anterior temporal cortex, previously not implicated in human eye movement control, supporting individual decisions where to look. This effect was largely driven by looking at faces. We compared similarity relationships between eye fixation patterns on the one hand and brain activity patterns on the other hand in humans. Searching for between-scene changes in activation patterns that mirrored changes in fixation patterns, we were able to identify the attention networks defined by previous experiments with artificial stimuli. In contrast, interindividual differences in fixation patterns, indicative of endogenous decisions about where to look, were mirrored by activation pattern differences in the orbitofrontal and anterior temporal cortex, particularly for scenes presenting faces. Thus, we could precisely replicate the role of the attention networks in a more natural viewing environment. Crucially, we also showed that previous lab experiments overlooked the importance of the orbitofrontal–anterior temporal network for individual differences in active vision. Our findings show that the orbitofrontal–anterior temporal network needs to be considered in studies of active vision, particularly in social situations.

## Significance Statement

Human attention networks have been replicated in a naturalistic setting. The attention networks could be mapped by comparing the similarity of fixation patterns and local brain activation patterns between scenes of a movie. In contrast, searching for brain activation reflecting interindividual differences in fixation patterns yielded an orbitofrontal–anterior temporal network. This network showed the highest similarity of fixation and brain activation patterns during scenes containing faces, in line with its known function for social communication.

## Introduction

The network of brain areas involved in guiding visuospatial attention has been in the focus of human neuroimaging research from early on (Corbetta et al., 1991, 1993). Although many details remain under active investigation, a broad consensus has been reached about a dorsal frontoparietal network that contributes primarily to overt and covert (with and without accompanying eye movements, respectively) shifts of attention, in close interaction with a ventral, dominantly right-hemispheric network including the temporoparietal junction area and posterior inferior frontal gyrus that supports reorienting attention to task-relevant stimuli (Pollmann and von Cramon, 2000; Wager et al., 2004; Corbetta et al., 2008).

These investigations mainly relied on variations of two experimental paradigms, the spatial cueing paradigm (Posner, 1984), and the visual search paradigm (Treisman and Gormican, 1988). The strength of these paradigms, illustrated by numerous experiments, is the high degree of control over stimulus parameters and task settings that can be achieved by using artificial displays with abstract items and simple task structures. In turn, however, the spatial cueing and visual search paradigms share the limitation that they create rather artificial task environments that are well-suited for isolated parameter variation but miss the complexity of the real world. For instance, human scene exploration can only to some degree be predicted by salience models of the environment (Betz et al., 2010). Where we look depends to a large degree on endogenous factors like the observer’s task set and expertise (Hayhoe and Ballard, 2005).

Here, we made use of a dataset that enabled us to investigate core aspects of the functional neuroanatomy of visuospatial attention in as rich an environment as can be investigated in an fMRI scanner: full-length movie viewing. Participants viewed the motion picture “Forrest Gump” while their brain activation (via the vascular BOLD response) was acquired in an fMRI session and their eye movements were recorded simultaneously (Hanke et al., 2014, 2016). The data is freely accessible via www.studyforrest.org.

Eye movements are a sensitive measure of attention shifts, because in natural viewing situations, the focus of attention is intimately linked to foveal vision. Attention can be displaced covertly during central fixation (Posner, 1984). However, when an observer makes a saccade, the focus of attention is linked to the saccade target location (Hoffman and Subramaniam, 1995; Deubel and Schneider, 1996; Guzhang et al., 2024). Thus, the gaze path is a good index of attention shifts during free movie viewing.

Brain imaging data obtained during movie viewing have been analyzed before. For instance, correlated changes in brain activation in posterior visual areas have been found in observers watching the same movie segments (Hasson et al., 2004). Naturalistic stimuli have also been shown to elicit widespread, content-specific activation patterns across the cortex—not only within the visual cortex but also in associative regions such as the dorsal stream and ventral temporal cortex (Haxby et al., 2020). In the present study, however, we used multivariate pattern analysis methods to relate eye movements to changes in brain activation patterns during movie viewing. Obviously, these two sources of data cannot be directly compared. Therefore, we used representational similarity analysis (RSA; Kriegeskorte et al., 2008) to compare the similarity of patterns within each dataset and to use these similarity relations as common ground for a comparison of fMRI and eye gaze data. This method allowed us to ask two questions: (1) In which brain areas does the variability of intraindividual between-scene activation patterns represent the variability of fixation locations between scenes? (2) In which brain areas does the variability of activation patterns between participants represent the between-participant (interindividual) variability of fixation locations?

The first comparison identifies areas that contribute to spatial attention shifts. Therefore, we expected a high correlation of between-scene similarity relations for BOLD activation patterns on the one hand and fixation density maps (FDMs) on the other hand in brain areas belonging to the dorsal and ventral attention networks of the brain.

The second comparison aimed to identify brain areas that support individual—implicit and explicit—decisions about where to look and what to attend to. In classical studies of visuospatial attention using the spatial cueing paradigm, this question was usually not addressed, because attention shifts were governed by clearly defined exogenous or endogenous cues. Therefore, it is less clear which brain areas would represent interindividual differences in gaze behavior during movie viewing, where participants' focus of attention may be influenced by a multitude of external and internal cues. One possibility would be that interindividual differences in eye movement patterns would be reflected by interindividual differences in the attention network. Alternatively, individual choices of eye movements may be driven by additional brain areas that reflect the viewer's interaction with the movie.

To preview our findings, we found the dorsal and ventral attention networks to support visuospatial shifts of attention, whereas the orbitofrontal–anterior temporal network reflected interindividual differences in fixation patterns, particularly while looking at faces.

## Materials and Methods

A custom analysis pipeline was developed for the analysis, which relied on the following packages: Nilearn (Abraham et al., 2014), NumPy (Harris et al., 2020), scikit-learn (Pedregosa et al., 2011), and a Python implementation of the RSA-Toolbox1 (Nili et al., 2014). Data management was realized through Datalad (Halchenko et al., 2021). All figures were created using the python packages matplotlib (Hunter, 2007), seaborn (Waskom, 2021), and surfplot (Gale et al., 2021). Surface visualizations were generated only for display purposes by projecting volumetric maps onto the fsaverage inflated surface using registration-based interpolation. To identify potential artifacts, we used MRIQC (Esteban et al., 2017).

### StudyForrest data and stimulus overview

The data analyzed in this study were obtained from different publicly available sources, mainly the StudyForrest project (Hanke et al., 2016). Fifteen volunteers (age 19–30, mean 22.4, 10 females) watched a slightly shortened audiovisual version of the feature film “Forrest Gump” (director: Robert Zemeckis, 1994) while functional images and their eye movements were recorded. All participants had previously participated in the initial phase of the StudyForrest data collection, where they listened to an audiobook adaptation of the film. The movie was divided into eight segments (runs) between 11 and 18 min. Participants were not given any instructions and could follow the movie according to their individual preferences. The movie was presented on a screen with a resolution of 720p at full width, situated at a viewing distance of 63 cm on a 1,280 × 1,024 pixel display. The number of volumes recorded per run fluctuated based on the film cuts, yielding different volumes for each run (451, 441, 438, 488, 462, 439, 542, and 338 volumes). The audio track was filtered to improve audibility in the noisy MRI scanner environment. Key noise frequencies at 690 Hz, 1,285 Hz, and their harmonics were attenuated to ensure clear audio delivery during echoplanar imaging. A distinctive feature of StudyForrest is the simultaneous acquisition of high-resolution eye-tracking data alongside functional MRI recordings.

### Functional data acquisition

MRI data were collected on a 3 Tesla Philips Achieva Stream scanner equipped with a 32-channel head coil. Functional volumes were obtained using a gradient echo sequence with 2 s repetition time (TR), 30 ms echo time, 90° flip angle, and a field of view (FOV) of 240 mm.

### Anatomical data preprocessing

The T1-weighted anatomical images were retrieved from Hanke et al. (2014). fMRIPrep utilizes Nipype (Gorgolewski et al., 2011) to integrate different preprocessing software packages. The T1-weighted (T1w) image was corrected for intensity nonuniformity with N4BiasFieldCorrection (ANTs version 2.3.3; Avants et al., 2011) and used as T1w reference throughout the workflow. The T1w reference was then skull-stripped with antsBrainExtraction.sh (ANTs, using the OASIS30ANTs as target template). Brain tissue segmentation of cerebrospinal fluid (CSF), white matter (WM) and gray matter (GM) was performed on the brain-extracted T1w with FAST (FSL 6.0.5.1; Jenkinson et al., 2012). Brain surfaces were reconstructed using recon-all (FreeSurfer 6.0.1; Dale et al., 1999; Fischl, 2012), and the brain mask estimated previously was refined with a custom variation of the method to reconcile ANTs-derived and FreeSurfer-derived segmentations of the cortical GM of Mindboggle (Klein et al., 2017). Volume-based spatial normalization to one standard space (MNI152NLin2009cAsym) was performed through nonlinear registration with antsRegistration (ANTs), using brain-extracted versions of both T1w reference and the T1w template. The following template was selected for spatial normalization: ICBM 152 Nonlinear Asymmetrical template version 2009c (Fonov et al., 2011).

### Functional data preprocessing

First, a reference volume and its skull-stripped version were generated using a custom methodology of fMRIPrep. Head-motion parameters with respect to the BOLD reference (transformation matrices and six corresponding rotation and translation parameters) were estimated before any spatiotemporal filtering using MCFLIRT (Jenkinson et al., 2002). BOLD runs were slice-time corrected to 0.971 s (0.5 of slice acquisition range 0–1.94 s) using 3dTshift from AFNI (Cox, 1996). The BOLD time series (including slice-timing correction when applied) were resampled onto their original, native space by applying the transforms to correct for head motion. The BOLD reference was then coregistered to the T1w reference using mri_coreg (FreeSurfer) followed by flirt with the boundary-based registration (Jenkinson et al., 2002; Greve and Fischl, 2009) cost function. Coregistration was configured with 6 df. The head-motion estimates calculated in the correction step were also placed within the corresponding confounds file. The confound time series derived from head-motion estimates and global signals were expanded with the inclusion of temporal derivatives and quadratic terms for each (Satterthwaite et al., 2013). All resamplings can be performed with a single interpolation step by composing all the pertinent transformations (i.e., head-motion transform matrices, susceptibility distortion correction when available, and coregistrations to anatomical and output spaces). Gridded (volumetric) resamplings were performed using antsApplyTransforms (ANTs), configured with Lanczos interpolation to minimize the smoothing effects of other kernels (Lanczos, 1964). Nongridded (surface) resamplings were performed using mri_vol2surf (FreeSurfer). Additionally, BOLD time series were resampled into MNI152NLin2009cAsym standard space to a 2 mm resolution, with no other modifications to the preprocessing pipeline.

### First-level analysis (scene transition)

The first-level general linear model (GLM) was implemented as a least-squares separate (LSS approach; Mumford et al., 2012) using Nilearn. With this approach, a separate GLM was calculated for each regressor of interest, which included all other events in the corresponding run as a separate nuisance regressor. Regressors of interest for the scene-transition analysis were specified as events occurring for 4 s following a change in scene setting or a change in the local setting (e.g., scene setting: school and local setting, school hallway or Principal Hancock’s office). All timings were based on annotations provided by Häusler and Hanke (2016). The LSS procedure resulted in 509 individual GLMs per participant. All regressors were convolved with a double-gamma hemodynamic response function. Additionally, each GLM incorporated 12 motion parameters (three for translation, three for rotation and their respective derivatives), the first five principal components from anatomical component correction (aCompCor; Behzadi et al., 2007), and a single regressor for CSF. Using the first five principal components from aCompCor is a well-established compromise between removing physiological noise and preserving neural signal. We did not uset tCompCor principal components because its variance-based component selection may capture stimulus-related neural activity in naturalistic movie-viewing paradigms, increasing the risk of removing signals of interest. An autoregressive noise model of order one was applied to account for temporal autocorrelation in the residuals. Slow signal drifts were modeled using a discrete cosine transform basis set. To eliminate the effects of low-frequency signal drifts, high-pass filtering with a cutoff of 100 s was employed. Furthermore, beta maps underwent slight smoothing using a Gaussian kernel with a full-width at half-maximum (FWHM) of 4 mm (Hendriks et al., 2017). Beta maps for the between-scene analysis were computed in native space using GM masks (threshold ≥ 0.2). For the between-participant analysis, all beta maps were subsequently registered to standard space (MNI152NLin2009cAsym).

### Eye-tracking data acquisition and preprocessing

Eye-tracking was performed using monocular corneal reflection and pupil tracking with an EyeLink 1000 eye tracker (software version 4.594) at a viewing distance of 63 cm. The temporal resolution of the eye gaze recording was 1,000 Hz. With an optical DVI extender, a time delay between video image and eye movement data of 100–133 ms was introduced [see Hanke et al. (2016) for a more detailed description]. To account for this delay, all eye movement data were normalized in time through a process that involved shifting the time series by the minimal video onset asynchrony of 100 ms. One participant was excluded from the eye movement analysis due to poor data quality (> 60% data loss).

### FDMs

For each 4 s scene, the corresponding eye movement data were plotted in an FDM of 1,280 width and 546 height (widescreen format, equivalent to a resolution of 720p). Prior to statistical analysis, the FDMs of each participant were read as grayscale images, a Gaussian filter with a sigma of 5 was applied, and the images were vectorized using NumPy (Fig. 1*A*1; Fig. 2*A*1). For the between-scene analysis, in each run, the vectors were compared pairwise across all available scenes using squared Euclidean distance. This approach resulted in individual eye movement representational dissimilarity matrix, whose shape depended on the number of available scenes (e.g., 59 × 59 for run-1; Fig. 1*A*1). The same FDMs and corresponding vectors were used for the between-participant analysis; however, the shape of the eye-related representational dissimilarity matrix was based on the number of participants, which was consistently 14 across all available scenes (Fig. 1*B*1). This analysis encompassed a total of 509 scene changes.

### Searchlight RSA and group-level analysis

We applied RSA (Kriegeskorte et al., 2008) to investigate the relationship between functional imaging data and eye movements in both between-scene and between-participant analyses. This was achieved by comparing the similarity of neuronal activity patterns (Fig. 1*A*2) across different scenes with the similarity of eye movement patterns during corresponding time intervals, using rank correlation (Spearman's rho). Spearman correlation was used because RSA primarily assesses the rank-order correspondence between RDMs rather than linear relationships. The analysis was performed across the brain using a searchlight segmentation algorithm (Kriegeskorte et al., 2006), dividing the brain into spherical clusters with a radius of 2 voxels (∼6 mm). A GM mask was applied to limit the analysis to voxels within GM, with no additional threshold imposed on the searchlights. To avoid spurious correlations, the lower triangle and diagonal of both dissimilarity matrices were excluded from the comparisons (Ritchie et al., 2017). For the between-participant analysis, functional data were transformed into MNI space prior to the computation of correlations; all subsequent analyses followed the same procedure as in the between-scene analysis (Fig. 3*A*2). Voxel wise effects were tested for reliability using permutation tests with the randomize function in FSL (5,000 permutations; Winkler et al., 2014) and threshold-free cluster enhancement (TFCE; Smith and Nichols, 2009). To enhance sensitivity to spatially extended effects, variance smoothing with a 6 mm FWHM was applied. The TFCE-based corrected voxel wise significance threshold was set at *p*_(FWE)_ < 0.05 for all statistical tests.

### Conjunction analysis

An additional searchlight RSA was conducted for each potential confounding parameter (visual and auditory), utilizing the same LSS beta maps. This analysis mirrored the procedure previously used in the between-scene analysis, substituting the FDM dissimilarity matrices with those derived from the respective feature. Finally, a conjunction analysis was performed to compare the permutation test results with the between-scene map, identifying voxels that were significant in both maps (Fig. 2).

### V1 model and structural similarity index measure (SSIM) RSA

To account for the influence of visual information, we computed additional RDMs based on the SSIM (Wang et al., 2004; Between-Scene ∩ SSIM) and the Deep Neural Network AlexNet (Krizhevsky et al., 2017; Between-Scene ∩ AlexNet Activation) as alternative similarity structures. For each 4 s scene, an average image was generated by combining all 100 frames (25 frames per second). These averaged images were subsequently used to compute SSIM scores and served as input to the AlexNet model. SSIM serves as a similarity measure that aligns with human visual perception, effectively quantifying perceptual differences between two images. Image similarity in SSIM is determined by a weighted combination of three comparison measures: luminance, contrast, and structure. To capture the correspondence between deep neural network representations and brain activity from low-level features (e.g., edges), we extracted feature representations from four layers of AlexNet (conv1, conv5, fc6, and fc7) using the ThingsVision toolbox (Muttenthaler and Hebart, 2021).

### Auditory feature RSA

The filtered audio track from the original data collection was extracted using the Python package and librosa (McFee et al., 2015) and decomposed into auditory features for each scene. The analyzed features included chromatic shift (Between-Scene ∩ Chromatic Shift), which measures changes in frequency content and highlights tonal or pitch variations over time, root mean square (Between-Scene ∩ RMS) energy, a measure of the signal's average power or perceived loudness; spectral bandwidth (Between-scene ∩ Spectral Bandwidth), which captures the range of frequencies present in a signal; and spectral centroid (Between-Scene ∩ Spectral Centroid), often associated with the perceived brightness of a sound and spectral contrast (Between-Scene ∩ Spectral Contrast), which measures the difference in amplitude to capture timbral characteristic.

### Eye movement parameter analysis

From the between-participant analysis, we identified three distinct regions of interest (ROIs): the orbitofrontal cortex as well as the left and right anterior temporal poles. All ROIs were extracted using Nilearn. The previously computed LSS native space beta maps were smoothed with a Gaussian kernel of 4 mm, transformed into MNI152NLin2009cAsym space and used to extract beta activity patterns. For each ROI, we compared neural representational geometries with multiple eye movement-derived models, including fixation duration, number of fixations, time series of the first two fixations, overall eye movement time series within a scene, and saccade amplitude. To obtain these parameters, in addition to the eye movement data, fixation and saccade events detected by the REMoDNaV algorithm in the StudyForrest dataset (Dar et al., 2021) were used. For each 4 s scene, fixation durations and saccade amplitudes were extracted as vectors, number of fixations was computed as a scalar, and 2D time series vectors were formed from *x* and *y* coordinates of eye movement data. Additionally, after excluding fixations <50 ms, time series of the *x* and *y* coordinates of eye movement data were extracted during the first two fixations as a 2D vector. Statistical significance was assessed using a permutation test, and results were corrected for multiple comparisons using the Benjamini–Hochberg procedure (*α* = 0.05).

### Face-sensitive ROI analysis

Events for the face and no-face analysis were again derived from openly accessible annotations (Labs et al., 2015) and Pliers (McNamara et al., 2017) using Google's Vision Face Extractor API. To maximize comparability, first-level LSS modeling employed the same hyperparameters as the scene-transition analysis. This procedure led to 643 face trials and 188 no-face trials per participant. Between-participant beta patterns extracted from the previously defined orbitofrontal cortex, left, and right anterior temporal pole ROIs were then used to compute representational similarities between vectorized FDMs and neural activity patterns for each ROI.

### Replication with new datasets

To validate the prior findings, we repeated the analysis on two independent datasets. Our aim was to identify datasets comparable to StudyForrest. While numerous naturalistic fMRI datasets have become publicly available in recent years, StudyForrest remains unique in combining naturalistic visual stimulation with simultaneous high spatial and temporal resolution eye-tracking.

### Stimulus overview and event segmentation

We selected a (1) published fMRI dataset of viewing a TV series that also featured rich audio–visual and narrative content (Chen, et al., 2017). From the original 22 participants (12 males, 10 females; age range 18–26 years, mean age 20.8 years), we analyzed the 16 participants publicly available. During the experiment, participants watched 48 min of the pilot episode of “Sherlock” (BBC). The audio–visual material was divided into two segments of 23 and 25 min, respectively. All participants were right-handed native English speakers with normal or corrected-to-normal vision. As no eye-tracking data were available, we estimated participants' gaze patterns using the DeepMReye software (Frey et al., 2021). Additionally, we analyzed (2) a subdataset from the naturalistic neuroimaging database, where 20 participants (10 males, 10 females; age range 19–53, mean age 25.8) watched the movie “500 Days of Summer” 2020. Scene annotations containing onsets or mainly faces were generated from data provided by the authors, plus additional sources (Born et al., 2023), and were further refined using the Python package Pliers (McNamara et al., 2017). Scenes with long shots, tracking shots, and sequence camera angles were excluded, as these focus mostly on the environment in the center of the image rather than on faces. Given that the Sherlock dataset includes only two runs and is considerably shorter than StudyForrest (eight runs), we shortened the scene duration from 4 to 3 s for the Sherlock analysis in order to increase the number of trials available for the RSA.

### Anatomical and functional preprocessing

Anatomical and functional preprocessing was again carried out with fMRIPrep (v21.0.2; Esteban et al., 2019), using a configuration similar to that applied in the prior analysis. Because DeepMReye requires all inputs to be in standard space, no computations were performed in native space; instead, we directly applied fMRIPrep to transform all data into the MNI152NLin2009cAsym template. In the Sherlock dataset, different participants had slightly different number of volumes. Therefore, the functional data were subsequently trimmed according to the instructions provided by the authors to ensure that all participants had the same number of TRs and were synchronized in time. Initially, participants were shown a brief diagram that was unrelated to subsequent stimulation. The corresponding images were removed from the analysis. The 500 Days of Summer preprocessing, however, was identical to StudyForrest.

### First-level analysis

To ensure the greatest possible comparability with the StudyForrest analysis, we again applied the LSS approach, estimating one GLM for each annotated scene or face onset in both datasets. All hyperparameters and confounding regressors from the initial first-level analysis were retained (smoothing, high-pass filter, aCompCor, etc.). Scenes containing prominently featured faces and scenes without any visible faces were identified using the available annotations together with automated feature extraction via pliers. Applying this procedure resulted in 78 face scenes and 39 no-face scenes in Sherlock, and 184 face scenes and 157 no-face scenes in 500 d of Summer.

### DeepMReye movement extraction

We extracted eye movement patterns in both datasets. Recordings of the eyes and their movements are often an incidental by-product of the selected FOV. Such data are frequently discarded during preprocessing or treated as nuisance signals. Recent advances in deep neural networks made it possible to estimate eye movements directly from this functional data in an additional preprocessing step. First, the brain is transformed into a standard space, and a mask is superimposed over both eyeballs. Second, the extracted signal from inside the mask is forwarded to the convolutional neural network (CNN). Third, in an optional step, gaze positions can be inferred at a temporal resolution higher than the TR of the imaging protocol. Since no eye-tracking data were collected in the original data collection, we were unable to train a model from scratch or retrain an existing model. For this purpose, Frey et al. (2021) provide several pretrained models which can be applied directly without adapting the CNN to the current dataset. Each decoding model was either trained on five independent datasets together or on one single dataset. Individual datasets emphasize different aspects of eye movements, from fixations, saccades, smooth-pursuit over visual search tasks, and free viewing. We opted for the model that was specifically trained for free-viewing data.

### Dissimilarity matrices

Although these approaches cannot match the precision of high-resolution laboratory eye trackers, they enable retrospective estimation of eye movements in datasets not originally collected for this purpose. The resulting eye movement data were mapped using the respective screen dimensions, smoothed with a Gaussian sigma of 20 mm, vectorized, and subsequently used as input for the RSA.

### ROI analysis and hierarchical Bayesian model

The ROIs identified in the previous analysis were reused to compare neural activity patterns with estimated eye movement patterns in face-dominant and face-absent scenes over all movies available. Due to unequal numbers of face and no-face scenes, we employed hierarchical Bayesian modeling with partial pooling at run level (nested within movies) to test whether the representational pattern similarity was different between the face and no-face condition and whether this pattern was stable across movies. For that, we used the brms package in R (Bürkner, 2017), which in turn uses the probabilistic programming language STAN (Carpenter et al., 2017), the tidyverse package (Wickham et al., 2019), bayestestR (Makowski et al., 2019), and estimated marginal means (Russell et al., 2018). Results were visualized using ggplot2 (Wickham, 2011). Measurement of uncertainty was incorporated via known standard errors at the run level. A random intercept for each movie-by-run combination was included to account for residual variability between individual segments and to enable partial pooling across runs and movies. We employed weakly informative priors for all fixed effects and for the standard deviation of random effects. Posterior inference was based on four Markov chains with 1,000 warm-up iterations and 4,000 sampling iterations to ensure convergence. To assess whether allowing the face effect to vary across movies would improve model adequacy, we compared models with and without a condition × movie interaction using leave-one-out cross-validation (LOO-CV). Predictive performance was quantified using the expected log pointwise predictive density (elpd), with differences in elpd indicating relative out-of-sample predictive accuracy.

### Data and code availability

All the necessary information for obtaining eye-tracking, functional and anatomical data can be found here: www.https://www.studyforrest.org/, https://www.naturalistic-neuroimaging-database.org/datasets, and https://openneuro.org/datasets/ds001132/versions/00003. The scripts for reproducing the analysis (including intermediate results) are located here: https://github.com/nimarek/study_gaze_tracks/.

## Results

### Brain areas representing between-scene fixation variability

We calculated an RSA to investigate where in the brain the dissimilarity of intraindividual between-scene brain activation patterns correlated with the dissimilarity of spatial fixation patterns between scenes of the movie.

We reasoned that brain areas that support shifts of spatial attention should show similar activation patterns between scenes in which participants would show similar spatial fixation patterns and dissimilar activation patterns if the fixation patterns were dissimilar. These similarity relations were observed in the areas of the attention network, particularly the frontal eye fields, along the whole extent of the intraparietal sulcus, in the ventral occipitotemporal cortex, the early visual cortex, and the inferior frontal gyrus (Fig. 1*B*1,2).

**Figure 1.**
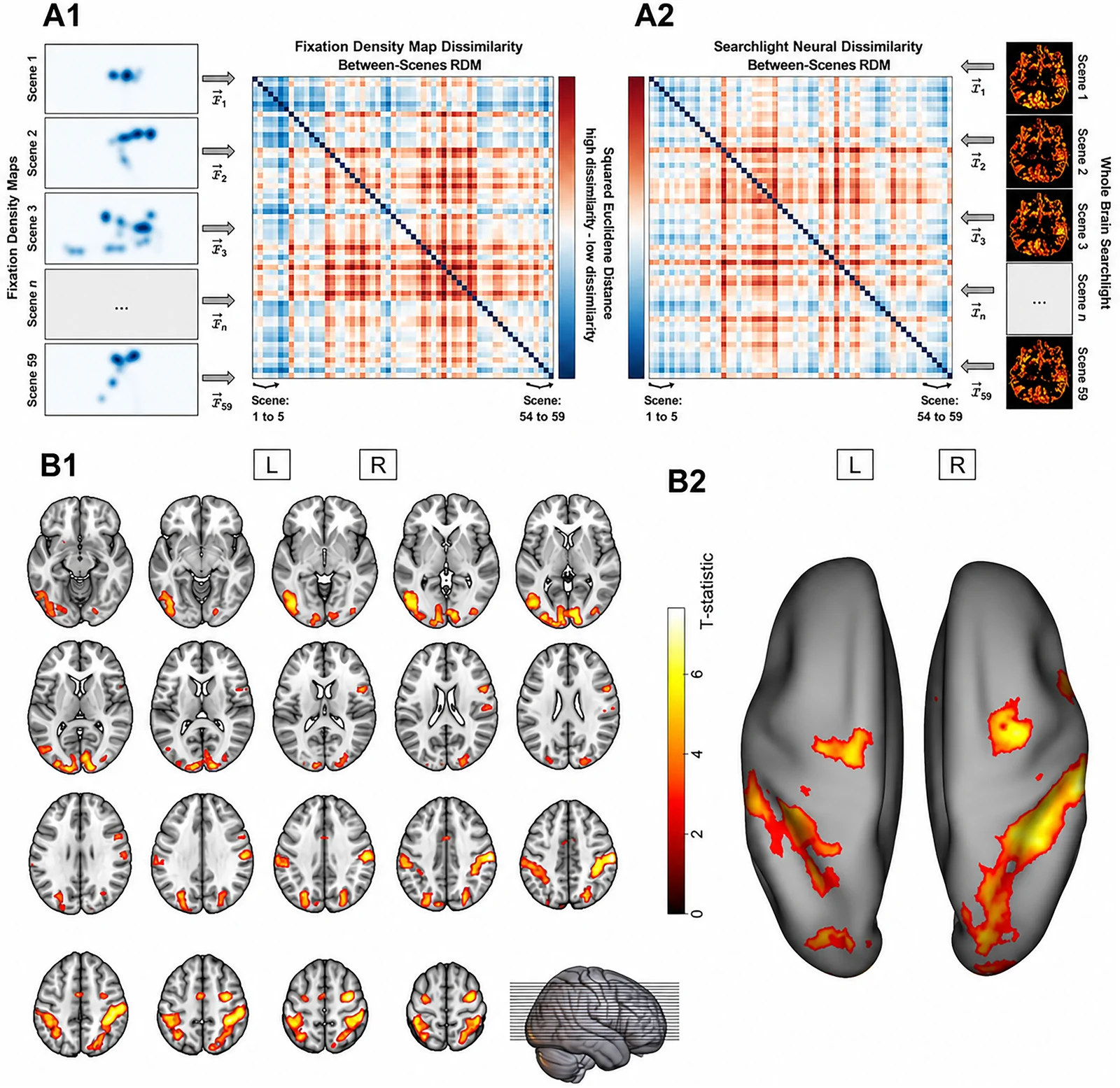
***A1***, Eye movement data from the initial 4 s period were visualized as FDMs for each participant and scene (e.g., depicted participant-02, run-01). Each FDM was then vectorized and compared with all other FDMs from this participant and the remaining scenes using squared Euclidean distance. To improve the visualization of the spatial distribution of eye movements, smoothing was applied with *σ* = 20, while a smoothing parameter of *σ* = 5 was used for the analysis. ***A2***, Least-square separate (LSS) beta maps for the corresponding period were computed in native space, utilizing individual whole-brain GM masks (threshold ≥ 0.2). LSS searchlight voxels were vectorized, and each vector was compared with those from all other scenes within the same run. The resulting between-scene representational dissimilarity matrix (RDM; ***A1***, right) was compared with the corresponding neural RDM (***A2***, left). This process was repeated for each voxel, producing a correlation map per participant and run. Spearman's *ρ* correlation maps were Fisher *z*-transformed, and significance was assessed using permutation tests with TFCE and familywise error (FWE) correction. ***B1***, Results of the searchlight-similarity analysis, showing clusters with significant correlations for between-scene comparisons. Neural representational dissimilarity matrices (RDMs) correlated significantly with RDMs derived from eye-tracking data in both the dorsal and ventral attention networks. ***B2***, Dorsal surface plot of the between-scene similarity results, showing a thresholded *t*-map.

However, scenes that differ with respect to spatial fixation patterns may also differ in visual characteristics. Particularly, the between-scene similarity of activation patterns may be due to the similarity of image structure between scenes, irrespective of whether these regions were attended. To exclude scene structure as a confound, we conducted additional analyses including measures of visual similarity to ensure that any observed effects were not solely driven by shared visual characteristics. To quantify scene structure, we used SSIM (Wang et al., 2004). To perform the control RSA for the between-scene analysis, we computed dissimilarity matrices based on SSIM and compared them with the between-scene neural RDMs to investigate brain regions responsive to similarities in scene structure. In an analogous control analysis, we investigated visual feature representations from the deep neural network AlexNet (Krizhevsky et al., 2017) using the ThingsVision toolbox (Muttenthaler and Hebart, 2021). The hierarchical structure of AlexNet allowed us to extract representations from different layers reflecting between-scene dissimilarity for increasingly abstract visual features. Importantly, both control analyses were performed using the exact same visual input that elicited the eye movements for the fixation-based analysis.

The movie contained not only visual but also auditory stimulation. Therefore, we also investigated dissimilarities of auditory structure between scenes of the movie as a potential confound of our fixation-related RSA. To this end, we used loudness (RMS), pitch and melody (chromatic shift), timbre (spectral bandwidth, centroid, and contrast), and the overall complexity of sound as separate parameters for computing dissimilarity matrices. Controlling for these auditory parameters was essential to ensure that neural patterns related to between-scene variability of fixation patterns were not confounded by auditory stimulation.

Conjunction analyses of the visual and auditory parameters with the between-scene gaze-brain similarity network are depicted in Figure 2. We found that, for visual parameters, only the representations derived from AlexNet, but not SSIM, showed an overlap with the between-scene gaze-brain similarity map from Figure 1 (correlations with SSIM were largely confined to the temporal cortex). Among the auditory parameters, chromatic shift, RMS (loudness), spectral bandwidth, and spectral contrast demonstrated overlaps. Notably, all these overlaps were restricted to visual areas within the occipital cortex, with no overlaps observed in frontal, parietal, or temporal parts of the between-scene gaze-brain similarity map.

**Figure 2.**
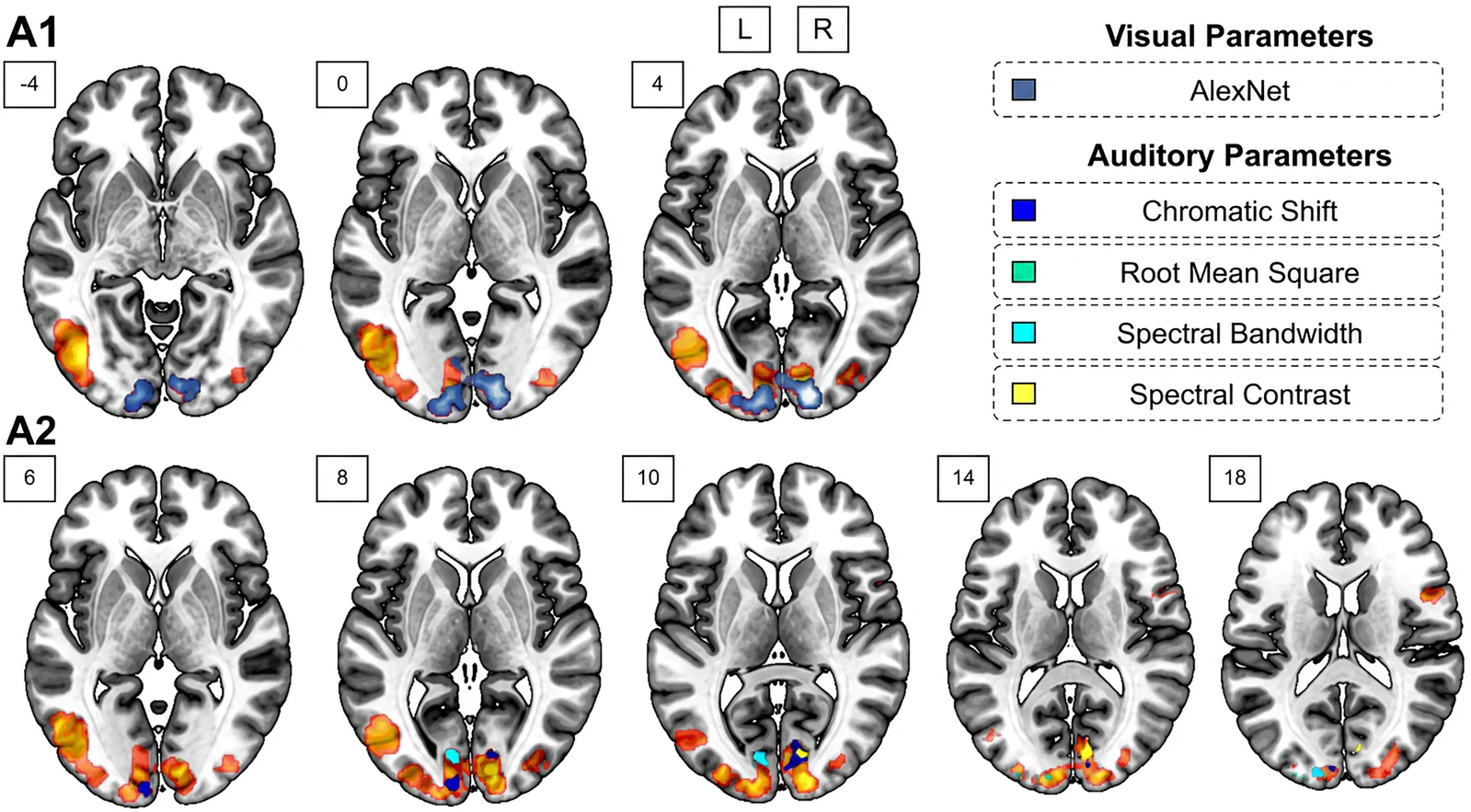
Pairwise conjunction analysis of the between-scene gaze-brain similarity network with visual as well as auditory scene parameters. Conjunction of visual and auditory parameters with gaze-related activation patterns was limited to the visual cortex. ***A***, Visual conjunction analysis: conjunctive representation of the between-scene gaze-related network (red-yellow, same as Fig. 1) with AlexNet representations. Conjunction analysis of between-scene gaze-related activation patterns with SSIM analysis yielded no significant conjunctions. ***B***, Auditory conjunction analysis: The conjunction analysis revealed that among the basic auditory features, only chromatic shift, root mean square (RMS), spectral bandwidth, and spectral contrast showed significant effects. Correlation maps and significance testing were computed following the same procedure outlined in Figure 1.

### Brain areas representing interindividual gaze variability

Our second question was which brain areas support individual choices where to look. Analogous to the analyses above, we again compared similarities between fixation patterns and BOLD activation patterns but this time between participants (Fig. 3*A*1,2). We reasoned that interindividual differences in fixation patterns, given identical stimulation, will reflect endogenous processes of fixation control.

**Figure 3.**
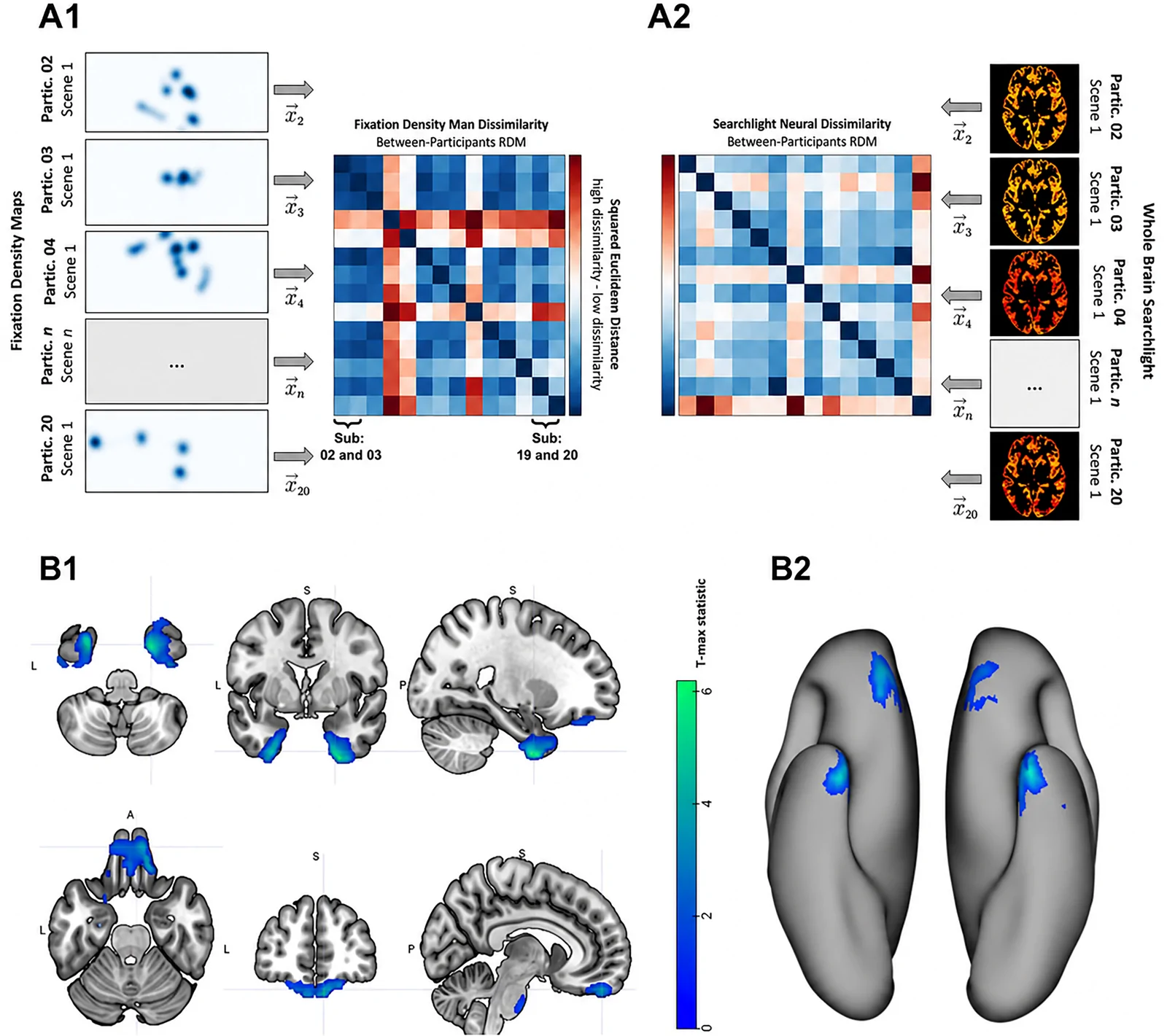
***A1***, The same FDMs used in the between-scene analysis were also applied to the between-participant analysis, comparing the spatial distribution of fixations across all participants within each scene. This process was repeated for all available scenes across all runs. ***A2***, LSS beta maps were transformed into MNI space, and pairwise dissimilarities between participants were computed within each scene using a group-level GM mask (threshold ≥ 0.2). The resulting between-participant representational dissimilarity matrix (RDM; A1, right) was then compared with the corresponding neural RDM (***A2***, left). All subsequent analyses followed the same procedure as described in Figure 1*B1*–*2*. Results of the between-participant analysis.

The interindividual dissimilarity of fixation patterns was reflected by the activation patterns bilaterally in medial orbitofrontal and medial temporopolar cortices and in anterior pons (Fig. 3*B*1,2). Thus, neural representations of interindividual fixation pattern differences were observed in areas distinct from the attention network. Orbitofrontal and anterior temporal cortices, linked by the uncinate fasciculus, are known to support social communication (Olson et al., 2013; Rouse et al., 2024).

Note that in this analysis, we analyzed variation between participants who were watching the same scenes, so potential stimulation confounds could not account for the results. It might be argued that different gaze paths go along with different visual input between participants. However, effects of differential retinal visual stimulation patterns would be expected particularly in early retinotopic brain areas, in which we did not detect activation pattern differences that were correlated with interindividual fixation pattern differences. Likewise, no such correlation was observed in higher visual areas with larger receptive fields in the ventral and lateral occipital cortex. Taken together, there is no evidence for a visual stimulation confound for the interindividual pattern differences observed in orbitofrontal and anterior temporal cortices. Note that comparable interindividual fixation pattern dissimilarities between one participant and two others do not imply that the latter two looked at the same locations. Thus, there is no simple correlation with visual stimulation.

Since the orbitofrontal and temporopolar cortices are not typically tied to eye movement control, we carried out ROI analyses in these areas to further analyze the characteristics of their gaze representation (Fig. 4). In each ROI, we performed RSAs comparing between-participant similarity in brain activity with that in various gaze parameters. We found the strongest correlation between fixation location time series and brain activation patterns. This is unsurprising, because this measure contained the same fixation locations used in the FDMs of the whole-brain analysis that defined the ROI, plus temporal information of the fixation sequence. These correlations should thus be seen as an upper limit of what can be expected from gaze versus brain activity correlations in these areas. On this background, it is remarkable that the restricted time series of the first two fixations in a scene yielded quite comparable correlations as the whole time series, at least in the orbitofrontal cortex and left anterior temporal cortex. More elementary gaze parameters—number of fixations, fixation duration, and saccade amplitude—also yielded significant correlations with brain activation patterns. Their absolute size, however, was much lower than the correlations with gaze time series, indicating that orbitofrontal and anterior temporal cortices represent complex fixation sequences more prominently than parameters of individual eye movements.

**Figure 4.**
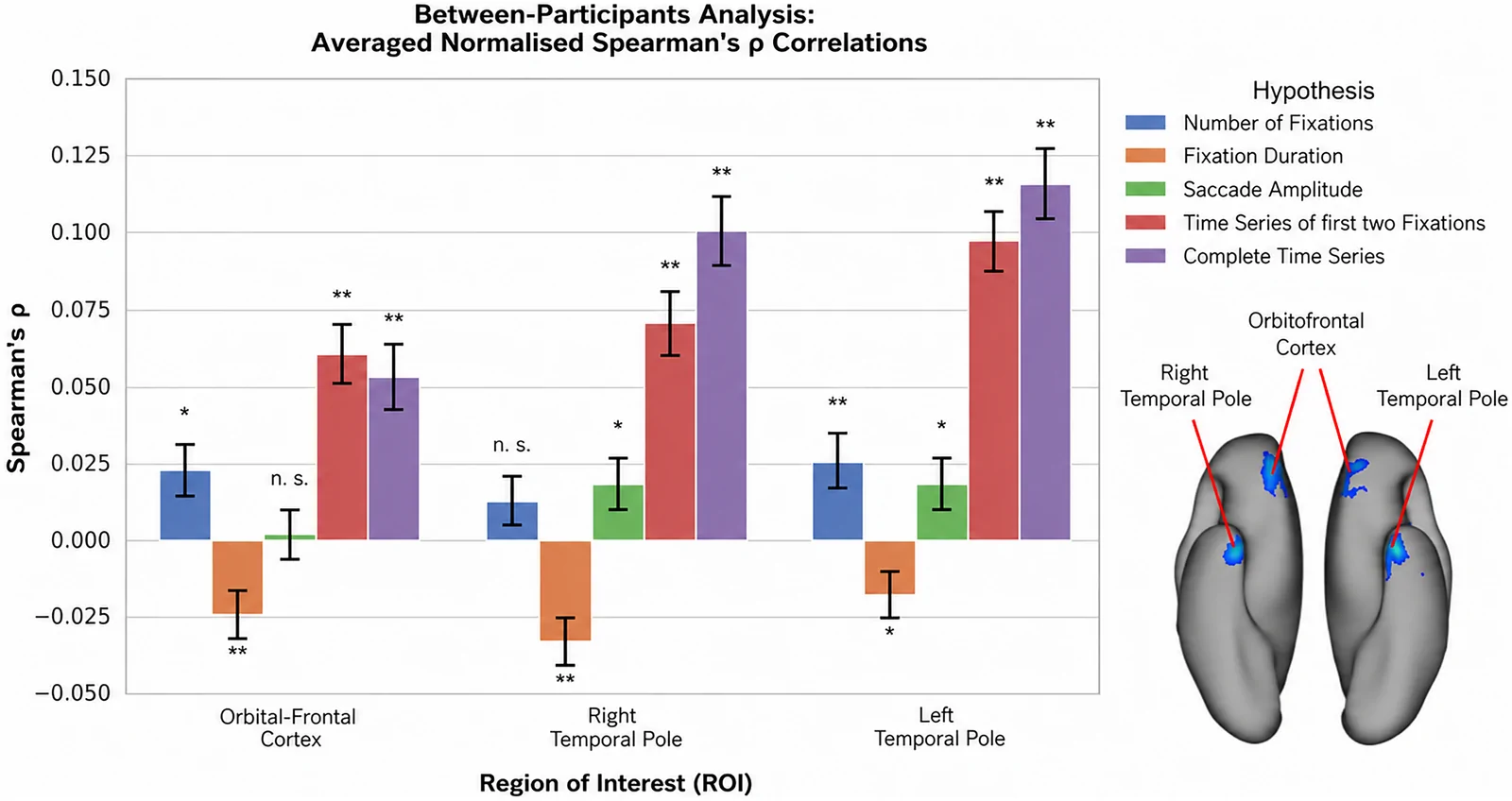
Average Spearman's correlation between BOLD activation patterns in the ROIs identified in the between-participant analysis and eye movement parameters. Strong correlations with brain activation patterns were found for fixation location time series, both for the complete 4 s time window and for the first two fixations alone. Significant but weaker correlations were found for basic gaze parameters such as number of fixations, fixation duration, and saccade amplitude. Statistical significance was assessed using permutation tests, and correction for multiple comparisons was performed using the Benjamini–Hochberg procedure with *α* = 0.05. Error bars depict the standard error of the mean.

### Selectivity for face viewing

The surprising involvement of the orbitofrontal–anterior temporal network, known to support social communication, made us consider what the social aspects of movie viewing might be. What comes to mind, particularly with a movie like Forrest Gump, consisting largely of dialogues, is face perception. Moreover, it is known that the anterior temporal cortex supports face perception, with lesions in the right anterior temporal cortex even leading to prosopagnosia (Evans et al., 1995; Gorno-Tempini et al., 2004; Thompson et al., 2004; Ding et al., 2020). The high similarity between the first two fixation locations and the activation patterns might also hint to a role of face perception, because the first two fixations are typically sufficient to recognize a face (Hsiao and Cottrell, 2008). In order to directly analyze the contribution of face viewing, we performed ROI analyses on orbitofrontal and anterior temporal cortices separately for scenes containing faces and scenes without faces (see Materials and Methods). This comparison yielded a high similarity for face scenes, but not for no-face scenes, suggesting that the overall similarity of interindividual fixation locations and activation patterns in these areas were largely driven by looking at faces in all three areas in the StudyForrest dataset (Fig. 5; see next paragraph for details).

**Figure 5.**
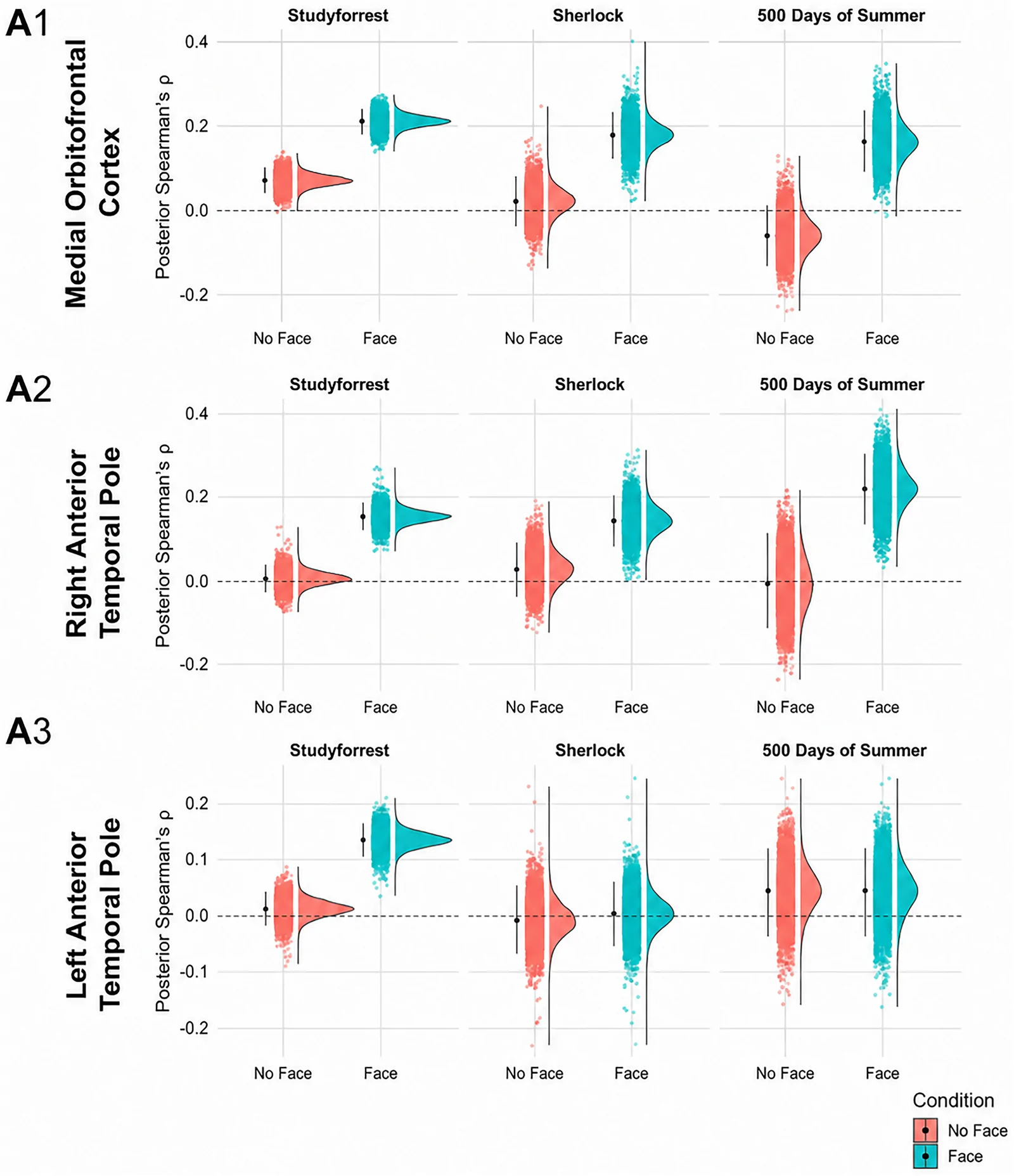
RSA results for the face and no-face conditions using the StudyForrest, Sherlock, and 500 Days of Summer datasets are shown for the ROIs: medial OFC (***A1***), right ATP (***A2***) and left ATP (***A3***). For StudyForrest, eye movement dissimilarities were computed using FDMs. For the other datasets, eye movement data were estimated from the functional images using DeepMReye (Frey et al., 2021), mapped to the respective screen dimensions, smoothed with a Gaussian kernel (*σ* = 20 mm), vectorized, and subsequently used as input for the RSA. ROI masks were defined based on the searchlight RSA results of the between-scene StudyForrest analysis. Each density plot depicts the posterior marginal distribution of the mean representational similarity (Spearman's *ρ*). Vertical lines indicate the 95% highest posterior density (HPD) interval around the posterior median (point). Across ROIs, hierarchical Bayesian modeling revealed higher representational similarity for face compared with no-face scenes, with robust face effects in the medial OFC and right anterior temporal pole, but a dataset-dependent pattern in the left anterior temporal pole with a reliable face effect was observed only in StudyForrest.

### Replication with independent datasets

Given the novelty of our findings of fixation pattern representation in orbitofrontal and anterior temporal cortices, we attempted a replication using two independent datasets. A notable challenge was the unavailability (to our knowledge) of another movie-viewing fMRI dataset that provided simultaneous eye movement recordings. We selected a dataset published by Chen et al. (2017), in which participants viewed the pilot episode of the TV series “Sherlock” (BBC). Additionally, we analyzed a second independent dataset from the Naturalistic Neuroimaging Database (Aliko et al., 2020), where participants watched the movie “500 Days of Summer.” To estimate fixation locations from the MR signal, we used the DeepMReye tool (Frey et al., 2021). Analogous to our previous analysis, we correlated between-participant dissimilarities in brain activation with (estimated) fixation locations, separately for face and no-face scenes. The analysis was carried out as an ROI analysis with ROIs defined by the orbitofrontal and anterior temporal regions obtained with the between-participant analysis of the StudyForrest dataset (Fig. 3). To account for unequal sample sizes between both movies and between conditions, we applied a hierarchical Bayesian model (Zhang et al., 2014; Bürkner, 2017; Marion et al., 2018) with partial pooling and likelihood-based weighting. In this framework, groups with less data contribute less strongly to parameter estimates, and conditions with more data contribute to the inference proportionally.

For the orbitofrontal cortex, posterior estimates from the hierarchical Bayesian model yielded consistently higher representational similarity for face compared with no-face scenes across all movies. The face–no-face contrasts (Δ_face − no-face_ = μ_face_ − μ_no-face_) and their 95% highest posterior density intervals (HPD_95%_) were positive in each dataset (StudyForrest: Δ_face − no-face_ = 0.140, HPD_95%_ = [0.132, 0.149]; Sherlock: Δ_face − no-face_ = 0.156 [0.132, 0.180]; 500 d of Summer: Δ_face − no-face_ = 0.222, [0.212, 0.231]), indicating statistically meaningful differences. Despite the consistent positive direction of the contrast, the magnitude of the face similarities varied across datasets. We compared models with and without face × dataset interaction using LOO-CV to assess whether the magnitude of representational similarity differed across datasets. Model comparisons are reported as expected log predictive density (Δ_elpd_). Here, larger values indicate larger differences in model fit relative to a model assuming equally strong similarities in all datasets. Δ_elpd_-values are interpreted relative to their standard error (SE), which reflect the uncertainty of the estimated difference in predictive performance (via LOO-CV). Model comparison using LOO-CV favored the face × dataset interaction model over a model without interactions (Δ_elpd_ = −125.9**,** SE = ± 74.3), indicating improved predictive performance when the face effect was allowed to vary between movies (Fig. 5). Following Sivula et al. (2025), we considered differences as reliable if the absolute value of Δ_elpd_ > 4 and larger than the associated SE. Thus, face scenes showed consistently higher representational similarities compared with no-face scenes, although the strength of the difference varied across datasets. The largest face versus no-face difference was observed in the 500 Days of summer dataset.

In the right anterior temporal cortex, posterior estimates from the hierarchical Bayesian model also indicated consistently higher representational similarity for face compared with no-face scenes across all movies (StudyForrest: Δ_face − no-face_ = 0.148, HPD_95%_ = [0.138, 0.158]; Sherlock: Δ_face − no-face_ = 0.116 [0.087, 0.146]; 500 d of Summer: Δ_face − no-face_ = 0.227, [0.144, 0.309]).

Again, these results indicate a robust face-related representational similarity in the right ATP in all datasets. In contrast to the medial OFC, LOO-CV provided little evidence that modeling the interaction improved predictive performance in the right ATP (Δ_elpd_ = −0.4, SE = ± 3.4), indicating comparable similarity across datasets.

In the left anterior temporal cortex, we did not observe a consistent face-related increase in representational similarity. Although the hierarchical model yielded a positive, overall face–no-face contrast when pooling across movies (Δ_face − no-face_ = 0.0453, HPD95% = [0.0359, 0.0545]), this effect was driven almost entirely by the StudyForrest dataset (StudyForrest: Δ_face − no-face_ = 0.1228, HPD_95%_ = [0.1147, 0.1411]), whereas in both the 500 d of Summer (Δ_face − no-face_ = 0.0004, HPD_95%_ = [−0.0099, 0.1411]) and the Sherlock dataset (Δ_face − no-face_ = 0.0126, HPD95% = [−0.0143, 0.0358]), differences between face and no-face scenes were at best small and statistically uncertain (HPD intervals overlapping with zero). The LOO-CV result (Δ_elpd_ = −255.2, SE = ±102.9) indicated that a model allowing the face effect to vary across datasets predicts the data better than a model assuming a common face effect across all movies.

In sum, these results indicate that representational similarity in the left anterior temporal pole does not show a consistent face viewing effect across datasets.

## Discussion

We compared spatial fixation patterns and brain activation patterns obtained simultaneously in an fMRI experiment to investigate the functional neuroanatomy of overt visuospatial attention in a complex realistic setting, namely, movie viewing. Between-scene variability of fixation patterns was mirrored by the variability of activation patterns in the areas of the attention network. In contrast, variability of fixation patterns between participants was reflected by brain activation patterns in orbitofrontal and anterior temporal cortices, areas not traditionally implied in eye movement control. Thus, these two networks appear to complement each other in that the attention network supports spatial shifts of eye movements, whereas the orbitofrontal/anterior temporal network is involved in the individual selection of where to look. The latter was observed mainly in scenes containing faces.

The first finding showed that the attention network, previously defined in experiments using the spatial cueing or visual search paradigms with abstract stimuli, also supports overt visuospatial shifts of attention in the much more complex and realistic setting of movie viewing. Note that we analyzed a wide range of visual and auditory parameters as potential confounds eliciting between-scene changes of activation patterns. These parameter changes, however, elicited overlapping activation with the fixation pattern network only in the early visual cortex. For visual features, activation of the visual cortex is unsurprising. Correlation of visual cortex activation with auditory features may not necessarily indicate auditory processing in visual areas but may be due to correlations between visual and auditory features in the movie (e.g., speech sounds occur during scenes with dialogues). Importantly, the observed activation patterns in the remaining network were not confounded with stimulation parameters and can be attributed to eye movements, i.e., overt attention shifts, during movie viewing. Thus, the essential role of the attention networks for visuospatial attention shifts, shown in a wealth of lab experiments, could be replicated by the analysis of shared similarities of fixation and brain activation patterns in naturalistic vision.

Activation patterns that mirrored the variability of spatial fixation patterns between participants, in contrast, were observed in orbitofrontal and anterior temporal cortices as well as in the ventral part of the pons, which is vital for the generation of horizontal eye movements (Strupp et al., 2011). Given that all participants received the same stimulation (the movie), interindividual differences in fixation patterns must be due to endogenous factors of the viewers, i.e., their individual—implicit or explicit—selection of fixation locations. This correlation of interindividual fixation patterns with brain activation patterns was particularly high for scenes containing faces in the orbitofrontal cortex and right anterior temporal cortex. We could replicate this pattern in two independent datasets. While the left anterior temporal cortex also showed a higher correlation between interindividual fixation patterns and brain activation in the StudyForrest dataset, this was not confirmed in the two other datasets. One reason for this inconsistency may be that eye movements were not directly measured in the replication datasets but estimated from the fMRI signal, inevitably leading to a loss of precision. However, this would not explain why consistent stronger correlations for face scenes were observed in orbitofrontal and right anterior temporal cortices, but not in the left anterior temporal cortex. The more consistent advantage for face-related scenes in right than left anterior temporal cortex may instead reflect a true processing difference, in line with neuropsychological evidence for more prominent right than left anterior temporal involvement in face processing, e.g., case reports of prosopagnosia following dominantly right anterior temporal atrophy (Gorno-Tempini et al., 2004; Thompson et al., 2004; Ding et al., 2020).

Orbitofrontal and anterior temporal cortices have traditionally not been linked to eye movements. For instance, orbitofrontal neurons responded to economic value independent of visuospatial factors, e.g., appearance of the reward on the left or right (Padoa-Schioppa and Assad, 2006). More recently, however, it was found that value signals of some orbitofrontal cortex neurons were amplified when the monkeys fixated near the valued item (McGinty et al., 2016) or that firing patterns depended on which attribute was fixated (Perkins and Rich, 2025). Furthermore, orbitofrontal neurons in macaques selectively responded to views of conspecifics compared with objects and to mutual gaze events (Dal Monte et al., 2022). Together with our finding of a higher correlation between fixation patterns and brain activation patterns in face than no-face scenes, this may indicate a specific role of the orbitofrontal cortex in eye movement processing that is not exclusive to but particularly strong during looking at faces. Moreover, electrical stimulation of orbitofrontal cortex neurons in monkeys reduced the distance of the monkey's fixation to a conspecific's eyes as well as the latency to reciprocate the other's directed gaze (Fan et al., 2024). This suggests even a causal role of the orbitofrontal cortex in eye movement control. One parallel to our study is that these monkey experiments have been carried out with freely viewing monkeys meeting actual conspecifics, i.e., a rich natural setting with social components, such as looking at other faces and gaze following, comparable to movie viewing.

Our data are inconclusive as to whether the representation of fixation patterns in orbitofrontal cortex and anterior temporal cortex serves active eye movement control or, rather, whether it is the consequence of specific eye movement patterns. In addition, it is not known how direct the link between neural processing in the orbitofrontal and anterior temporal cortex is. For instance, are there overlapping areas that are activated during looking at faces and during face-related eye movements, as in the fusiform face area (Wang et al., 2019)? Does this activity serve the perception of social–emotional face cues, as has been argued more generally for face-related fixation sequences by Su et al. (2025)? Or do similar eye movement patterns simply elicit similar social–semantic contents in this network? Given the above evidence, we tend to think that the orbitofrontal–anterior temporal network influences where we look in social situations, but this needs to be confirmed by further research, e.g., using transcranial magnetic or ultrasound stimulation to disrupt its function. If the orbitofrontal and anterior temporal network is involved in eye movement control, we think that it may create a priority map where to fixate, particularly during looking at faces. This map may then be communicated to the attention network that, in turn, prepares the actual eye movements.

The anterior temporal activation patterns reflecting between-participant variability of fixation patterns were observed dominantly in the medial and ventral part of the temporal pole, overlapping with area TGm according to Fan et al. (2014). Area TGm activity was found to be correlated with activity along the superior frontal sulcus, reaching back into the frontal eye fields (Fan et al., 2014). Due to this connectivity, area TGm may be the link between the orbitofrontal–anterior temporal network and the attention networks. This hypothesis, however, needs to be investigated further in future studies.

The orbitofrontal cortex and anterior temporal cortex have long been known to support social cognition (for recent reviews, see Olson et al., 2013; Herlin et al., 2021; Rouse et al., 2024). Lesions of the orbitofrontal cortex, often as part of the behavioral variant of frontotemporal dementia, may lead to apathy, disinhibition, or failed social norm compliance (Peters et al., 2006; Hornberger et al., 2011; O'Callaghan et al., 2016). The role of eye movements in this context has so far been largely neglected. However, movie viewing demands looking at the right places to pick up social communicative cues, e.g., for person identification, facial emotion expressions, but also lip reading, particularly during dialogues. When looking at faces, the first two fixations contain sufficient information for person recognition (Hsiao and Cottrell, 2008). In this context, it is instructive that the orbitofrontal and anterior temporal activation patterns were strongly correlated with the visuospatial fixation sequence even when analysis was restricted to the first two fixations. Processing face-specific eye movements may thus contribute to the known role of the orbitofrontal–anterior temporal network in face recognition indicated by the presence of “face patches” in both the anterior temporal lobe and orbitofrontal cortex (O'Scalaidhe et al., 1997; Tsao et al., 2008). In fact, a recent MEG study showed that face-related fixation sequences could be decoded from the human orbitofrontal cortex, even before the same sequences could be decoded from the anterior temporal cortex or more posterior brain areas (Su et al., 2025). This study suggested that the anterior temporal cortex received information about face-related gaze sequences from the orbitofrontal cortex, in line with models of social control exerted by the orbitofrontal cortex (Rouse et al., 2024). Our data may suggest this social control function includes eye movement control in social situations.

## Conclusion

We have shown that the dorsal and ventral attention networks on the one hand and the orbitofrontal–anterior temporal network on the other hand complement each other in representing eye movements in free viewing. While the attention networks known from artificial lab situations also support eye movements during natural looking, the anterior temporal–orbitofrontal cortex network complements the attention networks concerning individual choices—implicit or explicit—about where to look. The latter was observed particularly when participants were looking at faces. This is consistent with the social communicative role of the anterior temporal–orbitofrontal network, adding a new aspect, the representation of eye movements in social situations, to its functions.

## Synthesis

Reviewing Editor: Alexander Soutschek, Universitatsklinikum Wurzburg Decisions are customarily a result of the Reviewing Editor and the peer reviewers coming together and discussing their recommendations until a consensus is reached. When revisions are invited, a fact-based synthesis statement explaining their decision and outlining what is needed to prepare a revision will be listed below. The following reviewer(s) agreed to reveal their identity: NONE. Note: If this manuscript was transferred from JNeurosci and a decision was made to accept the manuscript without peer review, a synthesis may not be available.

The reviewers appreciate the authors' revisions, particularly the more explicit acknowledgment of alternative interpretations of the interindividual OFC/anterior temporal findings and the limits of the correlational analysis. They are satisfied that their main concerns have been adequately addressed and they have no further comments.
